# Primary Care Considerations for the Pediatric Endurance Athlete

**DOI:** 10.1007/s12178-024-09883-1

**Published:** 2024-01-30

**Authors:** Rhonda A. Watkins, Rafael Verduzco Guillen

**Affiliations:** 1 Department of Orthopaedic Surgery, Division of Pediatric Orthopaedics, University of California, San Francisco, San Francisco, CA USA; 2 Pediatric Orthopedics, UCSF Benioff Children’s Hospitals, 747 52nd Street, Oakland, CA 94609 USA; 3 School of Medicine, University of California, San Francisco, San Francisco, CA USA

**Keywords:** Mental health, Endurance athlete, Pediatric athlete, Overtraining

## Abstract

**Purpose of Review:**

This study aimed to provide an overview of some of the medical concerns surrounding the care of the pediatric endurance athletes and add to the limited literature specific to the pediatric endurance athlete.

**Recent Findings:**

Endurance athletes are at risk for overtraining, relative energy deficiency in sport (RED-S), overuse injuries, nutritional deficiencies, and sleep dysfunction. Youth runners and female endurance athletes are particularly high-risk populations for RED-S; nutritional deficiencies and their care should involve thoughtful mitigation of modifiable risk factors. The growing endurance athlete may experience slightly different cardiac adaptations than the adult endurance athlete with the long-term implications of these changes still unclear.

**Summary:**

Endurance sports are common among youth athletes. Multidisciplinary care that includes screening and early intervention for high-risk areas is critical to optimize their care and promote, safe lifelong sport participation.

## Introduction

Endurance sports such as running, rowing, swimming, and cycling are characterized by motions that involve repeated isotonic contractions of large skeletal muscle groups [1]. Despite an overall decline in high school sport participation since 2018, endurance sports remain popular among youth athletes. According to the National Federation of State High Schools Associations (NFHS) 2021–2022 participation survey, track and field was the most popular sport for girls and the second most popular for boys [2]. Cross country was fifth most popular for girls and fourth for boys while was swimming ninth and tenth, respectively, for girls and boys [2]. This amounts to over 1.7 million high school endurance sport participants yearly [2]. The top ten placement of several endurance sports among youth athletes warrants a deeper investigation of the care needs surrounding participation in such sports.

Training for an endurance sport usually requires long hours of intense year-round activity, which can often make it difficult to participate in other sports, lending itself to sport specialization and the associated increased risk for overuse injury [3]. In addition, participation often begins at a young age which may lead to early sport specialization. Running for example often starts in childhood, in part due to ease of access, relatively low cost of participation and parent participation that results in their kids participating in tow. These factors can lead to an increased risk for under fueling, overuse injuries, overtraining, and burnout. This is unfortunate as many endurance sports can be practiced for life and hence have the potential to foster physical activity participation and its many associated benefits beyond childhood.

Caring for the youth endurance athlete requires knowledge of their unique sporting demands as well as awareness of common injuries encountered by these athletes. Furthermore, knowledge of the youth athlete growth and developmental changes are essential as these often impact their injury risk. Literature specific to pediatric endurance athletes is limited. This paper will contribute to that void by highlighting some primary care considerations for pediatric endurance sport athletes.

## Cardiovascular Considerations

Regular, persistent exercise leads to predictable changes in the heart architecture that allows it to sustain the demands of increased cardiac output needed for activity. These changes can be seen on ECG (sinus bradycardia, large QRS voltages) and are considered normal in the athlete [4]. They reflect modest increases in left and right ventricular cavity size from both increased myocardial wall thickness and chamber size, higher vagal tone, increased cardiac output, and slowing of resting heart rate to allow more rapid heart rate recovery after exercise and improved diastolic function [5, 6••]. It has been reported that pediatric athletes experience these same exercise-induced cardiac adaptations as those seen in adults, but the long-term health implications of these findings are unclear [6••]. Other reports suggest that the cardiac hypertrophy is less pronounced in pediatric athletes, but as is the case with adults, the most pronounced cardiovascular adaptations appear in endurance sports like rowing, triathlon, and swimming [7•].

Exercise is an effective way to moderate overall cardiac risk, but in excess, the development of paroxysmal atrial fibrillation (AF) has been reported in adult long-term endurance athletes who exercise for 15–30 h per week for decades or longer [8]. A metanalysis found that long-term adult endurance athletes have an increased risk of developing lone paroxysmal atrial fibrillation compared with the risk for sedentary people, but the risk of stroke in athletes with AF is lower than that in age-matched non­athletes with AF [9]. This may have implications for youth endurance athletes as they age as this association of excessive endurance activity with AF seems to increase after 1500–2000 lifetime hours of sports practice [7•].

Sudden cardiac death (SCD) is the leading cause of sports-related death among competitive athletes [8]. While SCD is uncommon among youth endurance athletes, identification and management of underlying conditions that lead to SCD can be lifesaving. The most common underlying cardiovascular abnormalities seen in younger athletes are hypertrophic cardiomyopathy (HCM), coronary anomalies, arrhythmogenic right ventricular cardiomyopathy, myocarditis, and channelopathies [8]. We now know that these causes vary by age. A study on the incidence and etiology of sudden cardiac death in youth competitive athletes found that anomalous coronary arteries accounted for 28% of cases in middle school athletes, while cardiomyopathies accounted for 47% of cases in college and professional athletes [10]. It is imperative then that providers treating pediatric endurance athletes know how to screen for HCM and identify its associated electrocardiogram (ECG) changes which can include prominent *Q* waves, deep negative *T* waves, and increased *QRS* amplitude with associated ST changes. Furthermore, the workup of a middle school aged athlete with exercise-induced chest pain or syncope should exclude anomalous coronary pathology. Secondary prevention via emergency action plan awareness and training as well as access to automated external defibrillators is also critical for sideline emergencies.

## Mental Health Considerations

Youth participation in sports offers many physical benefits and has been thought to be a protective factor for mental health. However, the type of sport involvement matters. Individuals in team sports were found to have better mental health than those not participating in sports. Meanwhile, youth involved in individualized sports, such as running and swimming, were found to have greater anxiety and depression, withdrawal, social problems, and attention problems compared to those who did not participate in sports. This could be due to the significant stress of performing alone in front of family and coaches, while also internalizing poor outcomes since there’s no team to distribute some of the blame [11]. Pediatric endurance athletes face both physical and mental fatigue from balancing sports with school, tolling training, and the need to perform. These added stressors can psychologically manifest with symptoms of anxiety and depression. These mental health concerns can become amplified during adolescence; therefore, it is important to act early to prevent further progression. While socio-economic and parental relationships are environmental factors that cannot be changed, primary care providers should focus on teaching coping strategies, mindfulness, and stress reduction therapies. Education on symptoms of anxiety and depression, where to seek help, and creating opportunities to share those concerns can offer athletes a “how and when” to bring up their concerns [12]. Lastly, psychopharmacology is an option where medication can be used to treat symptoms while minimizing damaging side effects for athletes like drowsiness, weight loss, and cardiac problems [13••] .

Overtraining syndrome (OTS) is defined as a chronic fatigue that impacts an athlete’s psychological attention on their emotional, social, and psychological development both sports and non-sports related, during times of intense training [13••]. Endurance athletes may be at increased risk for overtraining syndrome due to their participation in intense training [13••]. Athletes may present with symptoms such as chronic muscle and/or joint pain, elevated resting heart rates, difficulty completing usual routines, sleep changes, and weight loss. However, overtraining has a real impact on the psychological wellbeing of an athlete and can present as personality/mood changes, fatigue, decrease academic performance, and lack of enthusiasm [14••]. The diagnosis of OTS is a diagnosis of exclusion as there are several other illnesses that can present similarly including asthma, anemia, hypothyroidism, immunodeficiency, and depression. Therefore, it is imperative that healthcare providers pay close attention to a patient’s history. Other labs that can help differentiate OTS from other illnesses are blood work, testosterone: cortisol ratio, overnight urinary cortisol: cortisone ratio, maximal heart rate at lactate threshold, and/or decrease maximal lactate concentration [15]. Recommended treatment involves limiting participation to a maximum of 5 days per week with at least 1 rest day, taking 2–3 months off per year, and participating in one sport per season with just one team [16].

Suicide ranks as the second leading causes of death in individuals ages 10–14, and ages 15–24, according to the Suicide Prevention resource center, but specific variation regarding pediatric athletes is not known. Furthermore, youth athletes are a unique subset of individuals with unique characteristics that make them susceptible to suicide ideation including a sense of perfectionism, fear of failure, and social cognitive theory within their respective sport, or the idea that an athlete immersed in a sports culture will acquire information about themselves based on those they interact with inside of the sport. It is important for a provider to understand the culture of their sport, any history of injuries that can be limiting their performance, relationship with family, and relationship with peers that can be added stressors on the athlete. Interventions such as cognitive behavioral therapy, transtheoretical model of change, motivational interview, mindfulness therapy, and psychophysiological therapies such as biofeedback heart rate variability (BFB HRV) have been proven effective at reducing suicidal ideation [17]. Prevention efforts should focus on working with coaches, caregivers, and the athlete to recognize and share feelings of suicide to get connected to providers and Lifeline centers. As well as bringing their attention back to enjoying a sport rather than over-emphasizing the result of their efforts.

## Sleep Considerations

Training for endurance sports usually requires a substantial time commitment that may not foster fulfillment of youth sleep recommendations. The competitive youth swimmer, for example, may practice 6 to 7 days weekly equaling 20 to 30 h per week [13••]. In addition to the time demands of endurance training, youth athletes must also balance the demands of school, homework, family, part-time jobs, and other social commitments. The American Academy of Pediatrics (AAP) recommends that children 6 to 12 years of age get approximately 9 h (h) to 12 h of sleep per and teenagers aged 13 to 18 years should sleep 8 to 10 h per 24 h [18]. Several studies have shown that most youth athletes do not meet the required sleep recommendations for their age groups [19, 20]. Many also function in a state of chronic sleep debt.

This is problematic because sleep is essential for injury prevention, recovery, and performance in athletes. Lack of sleep impairs cognition, learning and memory consolidation, mental well-being, disrupts growth and repair of cells, and metabolism of glucose and lowers the protective immune response to vaccination and resistance to respiratory infection [21]. A study of over 100 youth athletes showed that athletes who obtained less than 8 h of sleep/night were 1.7 times more likely to suffer an injury than those who slept 8 h or more [22]. An older study showed that sleeping only 6 or fewer hours was associated with increased fatigue-related injuries in their youth athlete population [23]. Important considerations for endurance athletes, as the literature suggests that athletes participating in endurance sports like running (68%) and swimming (63%), suffer overuse injuries more frequently than non-endurance sport athletes [24].

Literature on sleep and performance in youth athletes is limited but seems to suggest that increased sleep is associated with improved performance. One study of Division 1 collegiate swimmers, following increased baseline sleep recommendations of 10 h per night for 6 to 7 weeks, showed many surpassing long-standing personal, school, or national bests [25]. Specifically, they found athletes swam a 15-m sprint 0.51 s faster, reacted 0.15 s quicker off the blocks, improved turn time by 0.10 s and increased kick strokes by 5.0 kicks [25].

When counseling youth athletes about sleep, it is important to recognize that challenges to sleep vary between different sports; for example, early morning practices are engrained in swim culture [21]. It is also important to recognize that circadian rhythms differ across individuals, age groups, and training phase. For example, adolescents more frequently experience a delay in circadian rhythms due to a desire to push back time of sleep onset and prolong morning arousal [26]. This desire may be further compounded by longer late-night practices. Taking a sleep history with a sleeping tool such as the BEARS (Bedtime problems, Excessive daytime sleepiness, Awakenings during the night, Regularity and duration of sleep, and Snoring) or the Pittsburgh Sleep Quality Index and adding anticipatory guidance regarding sleep to routine visits can make a tremendous difference for youth athletes.

Some prudent recommendations for youth endurance athletes on sleep are: [25, 26]

1. Keep a regular sleep–wake schedule, going to bed and waking up at the same times every day, on weekdays and weekends.
2. Use bright morning light to help reset the teenage circadian rhythms to an earlier schedule.
3. Limit caffeine intake, especially in the afternoon and evening.
4. Maintain an optimal bedroom sleeping temperature (60–67 °F).
5. Extend nightly sleep for several weeks to reduce your sleep debt before competition.

## Nutrition Considerations

Nutrition is an essential part of the discussion when taking care of the youth endurance athlete. Recommendations for these athletes should focus on maintaining adequate carbohydrate, protein, and fat intake to support adequate energy availability for the growing and developing body. Attention to essential vitamins and minerals intake is also necessary. A pediatric athlete’s energy is provided through the consumption of the macronutrients: carbohydrate, fat, and protein. Unfortunately, many youth athletes do not meet their daily recommended nutrient needs which can put them at risk for injury and poor performance [27].

It is difficult to prescribe exact energy requirements for young athletes, but it is strongly recommended that they are not in a negative energy balance and have sufficient energy availability (EA) for growth. Recommendations for youth athlete daily energy intake is limited. Studies in young adult runners suggest an estimated daily requirement of approximately 2500–2800 kcal per day for female runners and 3100 to 3600 kcal/day for male youth runners [6••]. Specific recommendations however should be tailored to the athlete’s sport, volume, and intensity of exercise, anthropometric values, and growth parameters. Providers should refer to a nutritionist if low energy availability is suspected in endurance athletes for tailored care.

Carbohydrates are the main fuel source for athletes, providing the glucose needed for energy. For athletes 4–18 years, carbohydrates should represent 45 to 65% of their total caloric intake, protein 25–30% of caloric intake, and fat 10–30% [28]. High dietary carbohydrate intake for several days before competition (carbohydrate loading) can improve performance for exercise > 90 min in duration in adults, but this benefit is thought to be insignificant in the growing athlete [27, 29]. Some carbohydrate sources include whole grains, vegetables, fruits, milk, and yogurt. Young athletes can meet their daily protein needs by making sure to include a source of lean protein such as eggs, milk, yogurt, nuts, beans, tofu, chicken, or fish at each meal.

Other nutrients of interest include iron and vitamin D. Iron is essential for oxygen delivery to the tissues. Iron depletion can be seen in endurance athletes due to increased iron losses in urine, feces, sweat, foot strike from intense impact activity or from menstrual blood in the case of female athletes. Low iron can also be seen in athletes who do not consume meat. Hence, female athletes, vegetarians, and distance runners should be screened periodically for low iron with a serum ferritin, complete blood count with differential and iron studies. Vitamin D regulates calcium and phosphorus absorption and metabolism and plays a key role in maintaining bone health. Higher vitamin D levels higher have been reported in athletes who train outdoors than those in those who train indoors [30]. This reflects the role of sunlight exposure in vitamin D synthesis and bodes well for endurance athletes who train outdoors. Those who train predominantly indoors, however, are at risk for deficiency and should be screened to maintain vitamin D levels of 40 ng/ml or higher [31]. Dietary sources of vitamin D and iron should be encouraged due to improved bioavailability over nutritional supplements [13••].

Post workout fueling is particularly important for the recovery of an endurance athlete and should include both carbohydrate and protein sources. Such foods, for example, graham crackers with peanut butter and juice, yogurt with fruit, or a sports drink with fruit and cheese should be consumed within 30 min of exercise [28]. Post workout hydration is also essential for recovery. Following activity, athletes should drink enough fluid to replace sweat losses about 4 mL/kg after exercise [28]. Electrolyte drinks can also be helpful to replace sodium lost during endurance events via sweating.

## Endocrine Considerations

Relative energy deficiency in sports (RED-S) is a syndrome that affects all body systems due to an imbalance of caloric intake vs caloric expenditure during intense physical activity, like participating in endurance sports. This difference in energy can lead to the three associated conditions formerly known as the female athlete triad: an eating disorder, amenorrhea, and osteoporosis [32]. In 2014, the International Olympic committee recognized that RED-S was more than these three conditions broadening the definition to encapsulate problems with immunity, cardiovascular, protein synthesis, growth/development, psychological, and endocrine, as well as, broadening this syndrome to include males [33].

Literature suggests that age may modulate the effects of RED-S in an athlete; the longer an athlete is in an energy deficient state and participating in a stressful sport/societal environment, the more susceptible they become to RED-S [34]. In other words, the older an athlete, the higher the likelihood of RED-S presenting [34]. Special attention then should be paid to the youth athlete in order to identify risk factors for RED-S and prevent the progression of the energy deficiency. Furthermore, RED-S has been reported at higher levels in high school athletes than in similar aged groups who do not participate in sports [32]. Being in a state of low energy availability can have adverse performance consequences on an athlete including decreased muscle strength, decreased training response, decreased concentration, irritability, depression, and increased injury risk [33]. Due to the large number of health consequences caused by RED-S, symptoms can vary greatly making it imperative that a provider pays close attention to the patient’s history, presenting symptoms, and intensity of physical training.

In female athletes, amenorrhea caused by low energy reserves can lead to a low estrogen state, low bone mineral density, and increased risk for bone stress injuries [13••]. As such, physicians should pay extra attention to menses when evaluating for stress fractures in endurance athletes. These stress fractures can force an athlete out of training which can be damaging to a young athlete’s mental state, as well as increase their risk of developing osteoporosis later in life. All female athletes, especially endurance athletes, should be screened for RED-S [24]. The female triad coalition has a screening tool focused on identifying risk factors and screening for return to play; the screen focuses on dietary habits, BMI, menstrual irregularities, and history of stress fractures [32].

Male athletes, who participated in elite endurance training, were found to have hormonal alterations and lower bone density with these dysfunctions persisting for days after extensive endurance competitions [35]. Education offers the most preventative solution given that only 50% of coaches, physicians, and athletic trainers can identify the components of the triad, while only 19% of high school nurses can identify all three components [33]. Peer-led educational modules focused on eating disorders have been found to be effective at reducing RED-S by educating athletes, coaches, and caregivers [33]. Additionally, treatment should include a multidisciplinary team of a nutritionist, psychologist, and sport provider who can provide interventions to improve eating disorders, regulate menstruation, and promote healthy training before an athlete is cleared to return to sports [13, 24].

## Overuse Injuries

Due to the long hours of sport training and in some cases competition, endurance athletes are at increased risk for overuse injuries. Some of the more common overuse injuries seen in pediatric endurance athletes are stress fractures, swimmer’s shoulder, and various apophyseal injuries like Osgood Schlatter’s disease. Some factors that can mitigate their risk for such injuries have already been discussed but there seems to be an opportunity for providers to counsel on training volume. Currently, there is no consensus on the minimum age of participation for certain endurance sports like marathon running but it is clear; the desire to participate should come from the child, not the parents or coach and should focus on fun and skill rather than competition [13••].

## Conclusion

Endurance sport participation is common among pediatric athletes. These sports require significant time commitment for intense hours of training that can put the athlete at risk for overuse injury, depression, anxiety, and sleep deprivation. Optimization of their overall health should include considerations of their sleep, nutrition, and mental health needs as well as an awareness of their endocrine and cardiac risks. Much of the data on health optimization for endurance athletes is still based on adult literature but as these sports continue to become more popular in the pediatric population, age and development specific data will be important to optimize care for this population. A comprehensive medical team of a physician, physical therapist, nutritionist, athletic trainers, and coaches is needed to meet all the care needs of the pediatric endurance athlete and foster safe, continued sport participation for life.

## Data Availability

No datasets were generated or analysed during the current study.
